# Supplementary material for: Accurate Long-Read RNA Sequencing Analysis Reveals the Key Pathways and Candidate Genes under Drought Stress in the Seed Germination Stage in Faba Bean
Source: Int J Mol Sci. 2024 Aug 15;25(16):8875. doi: 10.3390/ijms25168875 (PMC11354372; doi:10.3390/ijms25168875)
Supplement: Supplementary file 1 [file ijms-25-08875-s001.zip › Supplementary Figures/figure captions.pdf]

Figure S1 The KOG functional categories.

Figure S2 GO classification of the faba bean unigenes.

Figure S3 KEGG pathway distribution of the faba bean unigenes.

Figure S4 The top 20 KEGG pathways enrichment with DEGs between E1 and C105 at 16 h (A) and 64 h (B) under drought stress.

Figure S5 PPI analysis of DEGs in comparison of T1_64 vs T2_64.

Figure S6 qRT-PCR validation of the DEGs in comparisons of T1_16 vs T2_16 and T1_64 and T2_64.
