# Supplementary material for: Accurate Long-Read RNA Sequencing Analysis Reveals the Key Pathways and Candidate Genes under Drought Stress in the Seed Germination Stage in Faba Bean
Source: Int J Mol Sci. 2024 Aug 15;25(16):8875. doi: 10.3390/ijms25168875 (PMC11354372; doi:10.3390/ijms25168875)
Supplement: Supplementary file 1 [file ijms-25-08875-s001.zip › Supplementary tables-revised/Table S1.pdf]

Table S1. Read number and length distribution after ISO-Seq analysis.

Subreads	Number
Subreads number	7530616
Average subreads length(bp)	3031
Max(bp)	171660
Min(bp)	50
N50	3573
CCS (Circular Consensus Sequence)	351263
5′-primer	208596
3′-primer	285767
Poly-A	176206
Full length	119989
FLNC (Full-length non-chimeric reads)	911363
Average flnc read length(bp)	2151
Transcript length interval	Number
<500bp	13502
500-1k	24053
1k-2k	23055
>2k	4736
Min Length (bp)	200
Mean Length (bp)	1019
Median Length (bp)	881
Max Length (bp)	7023
GC(%)	40.19
N50	1280
Total Transcipts	65346
Total Genes	65346
Total assembled bases(bp)	66603946
