# Supplementary material for: Accurate Long-Read RNA Sequencing Analysis Reveals the Key Pathways and Candidate Genes under Drought Stress in the Seed Germination Stage in Faba Bean
Source: Int J Mol Sci. 2024 Aug 15;25(16):8875. doi: 10.3390/ijms25168875 (PMC11354372; doi:10.3390/ijms25168875)
Supplement: Supplementary file 1 [file ijms-25-08875-s001.zip › Supplementary tables-revised/Table S10.pdf]

Table S10 Assay for the content of amino acids in AAT transient expressed tobacco

Amino acid content (ug g^{-1} FW)	0 d post drought			10 d post drought		
	Uninoculated	Empty vector	AAT	Uninoculated	Empty vector	AAT
Aspartate	48.38b	82.00b	148.90a	101.17a	118.13a	136.98a
Glutamate	208.85a	143.33ab	108.51b	15.88b	18.64b	38.96a
Serine	124.92 a	98.26 a	119.38a	100.09a	108.89a	98.65a
Glycine	18.36a	15.30 a	15.60a	40.69a	41.67a	31.45b
Histidine	38.40a	32.16a	38.67a	41.41a	36.89a	35.09a
Arginine	144.61b	63.97b	718.31a	881.47b	990.50a	760.23c
Threonine	58.26a	39.12b	43.75b	72.56a	84.14a	72.27a
Alanine	64.19a	55.37a	68.03a	104.36a	134.01a	128.47a
Proline	76.57b	39.14b	318.12a	1372.87ab	1725.65a	961.33b
Tyrosine	35.56a	24.68b	21.31b	60.46a	60.40a	55.46a
Valine	58.86a	32.00b	31.05b	81.25ab	85.79a	70.48b
Methionine	6.99a	6.72a	8.22a	26.02a	29.04a	23.96a
Cysteine	3.17c	5.22a	4.49b	4.58b	4.05b	9.21a
Isoleucine	59.37a	61.68a	62.29a	109.09b	107.28b	127.65a
Leucine	87.66a	55.71b	59.37ab	182.25a	191.74a	187.89a
Phenylalanine	43.48a	32.33a	33.96a	109.37a	107.87a	100.90a
Lysine	113.70a	77.82b	78.01b	150.25a	193.58a	175.02a
