# Supplementary material for: Accurate Long-Read RNA Sequencing Analysis Reveals the Key Pathways and Candidate Genes under Drought Stress in the Seed Germination Stage in Faba Bean
Source: Int J Mol Sci. 2024 Aug 15;25(16):8875. doi: 10.3390/ijms25168875 (PMC11354372; doi:10.3390/ijms25168875)
Supplement: Supplementary file 1 [file ijms-25-08875-s001.zip › Supplementary tables-revised/Table S11.pdf]

Table S11 Primers used in present study

Gene	Forward primer sequence(5'-3')	Reverse primer sequence(5'-3')
**qRT-PCR**		
TRINITY_DN21706_c0_g1	GACTTGCATTCTCAGCGACA	TTGCGCCCATAATAAGAACC
TRINITY_DN28287_c0_g1	GCCCTATTTGAATGATGCTCTCGTGA	CTGGTTGGTTTTCTTCGCTTGCTTGT
TRINITY_DN26381_c0_g1	AGAAGAGTTGTTGGAGTTAGGA	TGGAGTAGAGACTGGCGTTG
TRINITY_DN21391_c0_g1	TCGGATTCCAGACTTTGCTTCTCT	GCTGTAGTTCTTCATAAACCTCAT
TRINITY_DN29210_c0_g1	ACAATCCAAATCCCACACAACGA	ATCAAGTGCGAGTGCATAACCAG
TRINITY_DN10493_c0_g1	TCCTGCTTCTACTTTTCCTCC	AACCATAACCCTTGATTGTGC
TRINITY_DN11206_c0_g1	GGTTTTGGTGACCCTTTTGTT	CCCCATAGGAGGAGTTTTAGC
TRINITY_DN19804_c0_g1	TCCAAAGTCGGTTCAAATCC	GTCGATATCTGCAGGGTCGT
TRINITY_DN13680_c0_g1	CTGGGAGGCTTCAGAGTTTG	CATTCCATGCTCCACAACAC
TRINITY_DN31004_c0_g1	GGTGCTGGTGATTCCTTTGT	CTTGGGAAGAGCAGGAATTG
TRINITY_DN6027_c0_g1	CTGCACACTTCTTGCTCAGAGT	GCCCACATTCTGTCAGTAATCA
TRINITY_DN5157_c0_g1	GCTTCACCTCATCTGGAAGG	ATCGAACTCAGTGGGGTGTC
TRINITY_DN8533_c0_g1	CCGATTCGTTTCCCTCTTTAC	GAGAGTGTCAGAAGCCATTCG
TRINITY_DN7026_c0_g1	TGGTGCTCTTTTGCTTTGTG	CTCAGGCTGACCCTTCTTTG
TRINITY_DN7405_c0_g1	GGTAGGGAGAGCCCACTTTACT	CAGCACCAGTCTCAGCAATAAC
TRINITY_DN7416_c0_g1	TTCCATCTCAATCCCTCTGG	CTTTTGGCTGCTTGCCTAAC
TRINITY_DN25802_c0_g1	CGCCACAGATTGTCCTAACAT	GTTGAGAACTTCACGCGCTAC
TRINITY_DN8484_c0_g1	CACTACTTTGGCGTTTTCGAC	GCTGTTCCATTTCTGCACTTC
TRINITY_DN8046_c0_g1	TGAAGAATTGCAGCCGTATG	TGGCAACTCTTTGCTGTTTG
TRINITY_DN14639_c0_g1	CCTGATGTCAACCATGCAAC	TGGTCACAAGCAGCACTAGC
TRINITY_DN16771_c0_g1	CGAGATGCTCGACCTTCAAC	AGAGAGCGAGGTTCCTCTGG
TRINITY_DN3638_c0_g1	GTCATCTTTCTCCGGCAATG	GAGAAGTGCCAGTGGCTGAC
TRINITY_DN39123_c0_g1	CTACCACCGATTCGCTCTTG	CGGCACTCCAATTTTCTCAG
TRINITY_DN25373_c0_g1	GCATGAAACATGCATCTGGA	AGATTCCATCCATGCACACC
TRINITY_DN7882_c0_g1	CCTTTCTCATTGGTTATGGGCT	ACGGCTTCAGGATTTTTGGATC
TRINITY_DN1742_c0_g1	TCTAAGTGGAGGGGAAAAAGGA	ACTCGAGCTGCAACGTGGAAAC
TRINITY_DN1271_c0_g1	CTTTTCCCTGCTGGGCTCTATT	TAAGGTTTGGCTTTTTGCTCGC
TRINITY_DN11712_c0_g1	CCTAACACTGGACTACTGACCG	AGAAACAGAAACCGCACGCAAT
TRINITY_DN3493_c0_g1	TAGCGCAGAGAACGAACCTT	CAACTGAAGAGCCACCGAAT
TRINITY_DN14525_c0_g1	CATCTCCAAATTTACGCAGTTGAC	TAATTTGAATGGTGGACGGTGTAG
TRINITY_DN8452_c0_g1	GGGCGAACTGAGACAAACACTG	ACAACGCACTCCCAACATAACC
TRINITY_DN19743_c0_g1	GGAAAGGAGTTTTGTTTGACCA	AGTAGCACCAGCACCAATGAGC
TRINITY_DN13709_c0_g1	AGCCAAAATCATCACTAACGAC	ACACCAAGCACCACTTCTCCAT
TRINITY_DN9688_c0_g1	TGGCTCGTGATACGGTGAAG	CGCCTACAGTGTCGGGTATG
TRINITY_DN6129_c0_g1	GGTGACACATTATTTGGGAGGA	GGTGTTTTGTAGGCTCTTGTGG
TRINITY_DN11468_c0_g1	CCACCAGAGAAGGACTCGAC	TGACACTTCTGCAAGGCAAC
TRINITY_DN25940_c0_g1	CGTTGTCAAGTCCGAGATTCA	AGGTTCCTTAGCAGCAACAGC
NADHD4	AGGGTTAGTGAGCACCATGC	ATAGCCAAAGGGAATACGCC
**Ectopic expression**		
AAT (TRINITY_DN8046_c0_g1)	CgACgACAAgACCgTgATGGCTTCTTCAGTACTCTCTG	gAggAgAAgAgCCgTCAGCTGACATTATGATAAG

**RT-PCR**		
AAT	TGTGCTCATAACCCTACCG	CCAATCCTTTCCGCTCCC
Actin	CCCTCCCACATgCTATTCT	AGAGCCTCCAATCCAGACA
