# Supplementary material for: Accurate Long-Read RNA Sequencing Analysis Reveals the Key Pathways and Candidate Genes under Drought Stress in the Seed Germination Stage in Faba Bean
Source: Int J Mol Sci. 2024 Aug 15;25(16):8875. doi: 10.3390/ijms25168875 (PMC11354372; doi:10.3390/ijms25168875)
Supplement: Supplementary file 1 [file ijms-25-08875-s001.zip › Supplementary tables-revised/Table S2.pdf]

Table S2 BUSCO analysis of transcript completeness.

BUSCO results	Full-length after corrected	
Complete BUSCOs	824	57.20%
Complete single-copy BUSCOs	365	25.30%
Complete Duplicated BUSCOs	459	31.90%
Fragmented BUSCOs	63	4.40%
Missing BUSCOs	553	38.40%
Total BUSCO groups searched	1440	100%
