# Supplementary material for: Accurate Long-Read RNA Sequencing Analysis Reveals the Key Pathways and Candidate Genes under Drought Stress in the Seed Germination Stage in Faba Bean
Source: Int J Mol Sci. 2024 Aug 15;25(16):8875. doi: 10.3390/ijms25168875 (PMC11354372; doi:10.3390/ijms25168875)
Supplement: Supplementary file 1 [file ijms-25-08875-s001.zip › Supplementary tables-revised/Table S4.pdf]

Table S4. Annotation statistics

	Unigene number (%)
NR	7,341(57.53)
Swiss-Prot	331(2.59)
KOG	7,139(55.95)
GO	4,702(36.85)
KEGG	7,616(59.69)
Total_annotation	7946(62.23)
Total	12, 760 (100)
