# Supplementary material for: Accurate Long-Read RNA Sequencing Analysis Reveals the Key Pathways and Candidate Genes under Drought Stress in the Seed Germination Stage in Faba Bean
Source: Int J Mol Sci. 2024 Aug 15;25(16):8875. doi: 10.3390/ijms25168875 (PMC11354372; doi:10.3390/ijms25168875)
Supplement: Supplementary file 1 [file ijms-25-08875-s001.zip › Supplementary tables-revised/Table S5.pdf]

Table S5 Reads statistics of Illumina sequencing

| Sample name | Total raw reads(M) | Total clean reads(M) | Total clean base(G) | Q20 | Q30 | Clean reads ratio(%) | GC content (%) |
|---|---|---|---|---|---|---|---|
| CK1_16_1 | 83.75 | 81.5 | 12.56 | 97.55 | 93.16 | 97.31 | 44.51 |
| CK1_16_2 | 82.77 | 80.6 | 12.42 | 97.55 | 93.15 | 97.38 | 44.01 |
| CK1_16_3 | 83.3 | 80.94 | 12.49 | 97.48 | 93.01 | 97.17 | 43.25 |
| CK1_64_1 | 83.54 | 81.46 | 12.53 | 97.54 | 93.05 | 97.51 | 43.48 |
| CK1_64_2 | 95.03 | 93.23 | 14.25 | 98.2 | 94.63 | 98.11 | 42.55 |
| CK1_64_3 | 82.27 | 80.27 | 12.34 | 97.83 | 93.86 | 97.57 | 43.74 |
| CK2_16_1 | 82.97 | 80.84 | 12.45 | 97.75 | 93.67 | 97.43 | 43.86 |
| CK2_16_2 | 83.82 | 81.78 | 12.57 | 97.82 | 93.78 | 97.57 | 44.42 |
| CK2_16_3 | 84.61 | 82.62 | 12.69 | 97.79 | 93.67 | 97.65 | 43.67 |
| CK2_64_1 | 87.45 | 84.91 | 13.12 | 97.63 | 93.48 | 97.1 | 43.83 |
| CK2_64_2 | 84.62 | 82.01 | 12.69 | 97.51 | 93.27 | 96.92 | 43.38 |
| CK2_64_3 | 82.29 | 80.02 | 12.34 | 97.66 | 93.53 | 97.24 | 43.76 |
| T1_16_1 | 82.61 | 80.43 | 12.39 | 97.5 | 93 | 97.36 | 44.66 |
| T1_16_2 | 82.9 | 80.65 | 12.44 | 97.51 | 93.06 | 97.28 | 44.52 |
| T1_16_3 | 83.79 | 81.39 | 12.57 | 97.3 | 92.61 | 97.14 | 44.43 |
| T1_64_1 | 85.02 | 82.44 | 12.75 | 97.43 | 92.99 | 96.97 | 43.67 |
| T1_64_2 | 83.69 | 81.37 | 12.55 | 97.55 | 93.17 | 97.22 | 43.33 |
| T1_64_3 | 86.01 | 83.7 | 12.9 | 97.47 | 92.94 | 97.32 | 44.08 |
| T2_16_1 | 80.13 | 78.1 | 12.02 | 97.72 | 93.58 | 97.46 | 43.16 |
| T2_16_2 | 80.15 | 78.02 | 12.02 | 97.81 | 93.85 | 97.34 | 43.64 |
| T2_16_3 | 83.49 | 81.01 | 12.52 | 97.56 | 93.32 | 97.02 | 44.21 |
| T2_64_1 | 85.53 | 83.41 | 12.83 | 97.79 | 93.72 | 97.52 | 43.91 |
| T2_64_2 | 83.2 | 81.22 | 12.48 | 97.82 | 93.76 | 97.63 | 43.53 |
| T2_64_3 | 81.29 | 79.52 | 12.19 | 97.92 | 93.98 | 97.82 | 43.46 |
