# Supplementary figures and images for: Accurate Long-Read RNA Sequencing Analysis Reveals the Key Pathways and Candidate Genes under Drought Stress in the Seed Germination Stage in Faba Bean
Source: Int J Mol Sci. 2024 Aug 15;25(16):8875. doi: 10.3390/ijms25168875 (PMC11354372; doi:10.3390/ijms25168875)

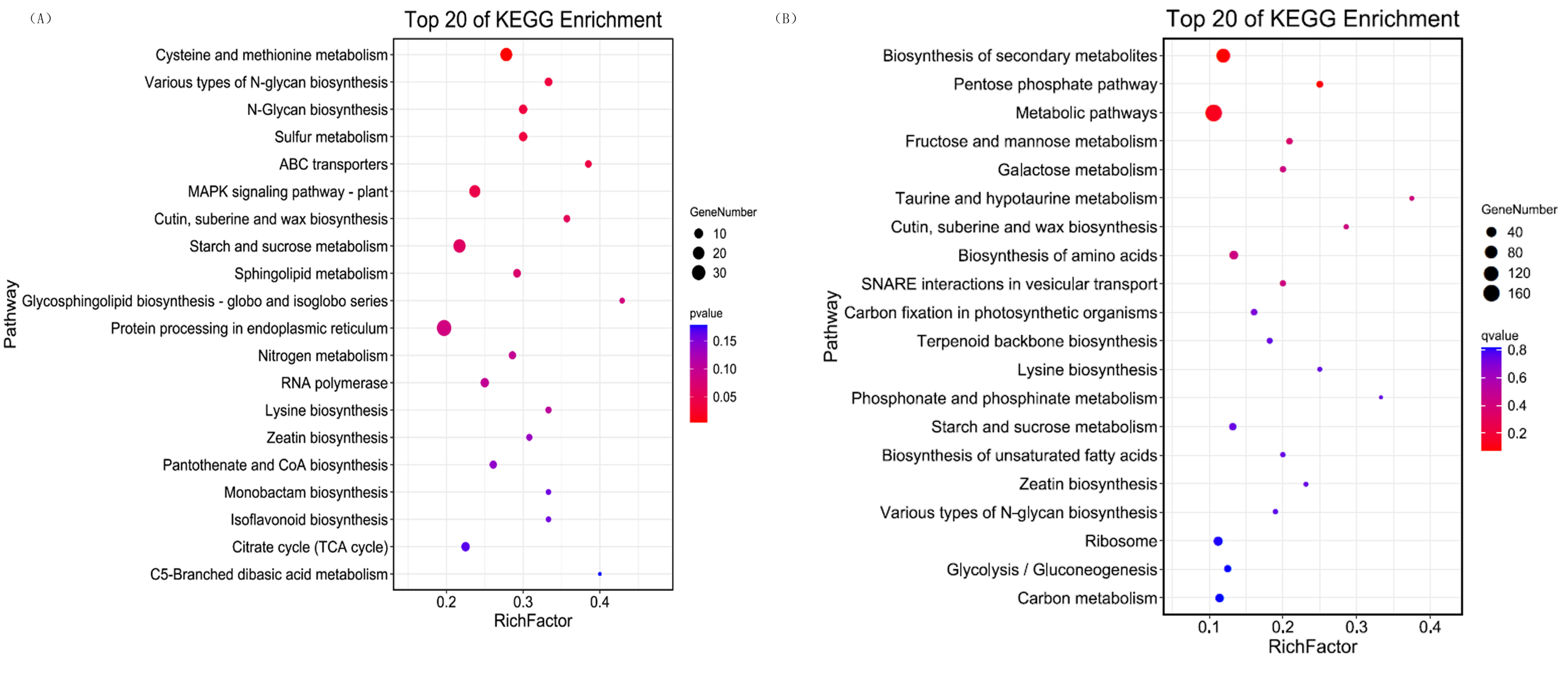

Supplement: Supplementary file 1 [file ijms-25-08875-s001.zip › Supplementary Figures/Fig S4.jpg]

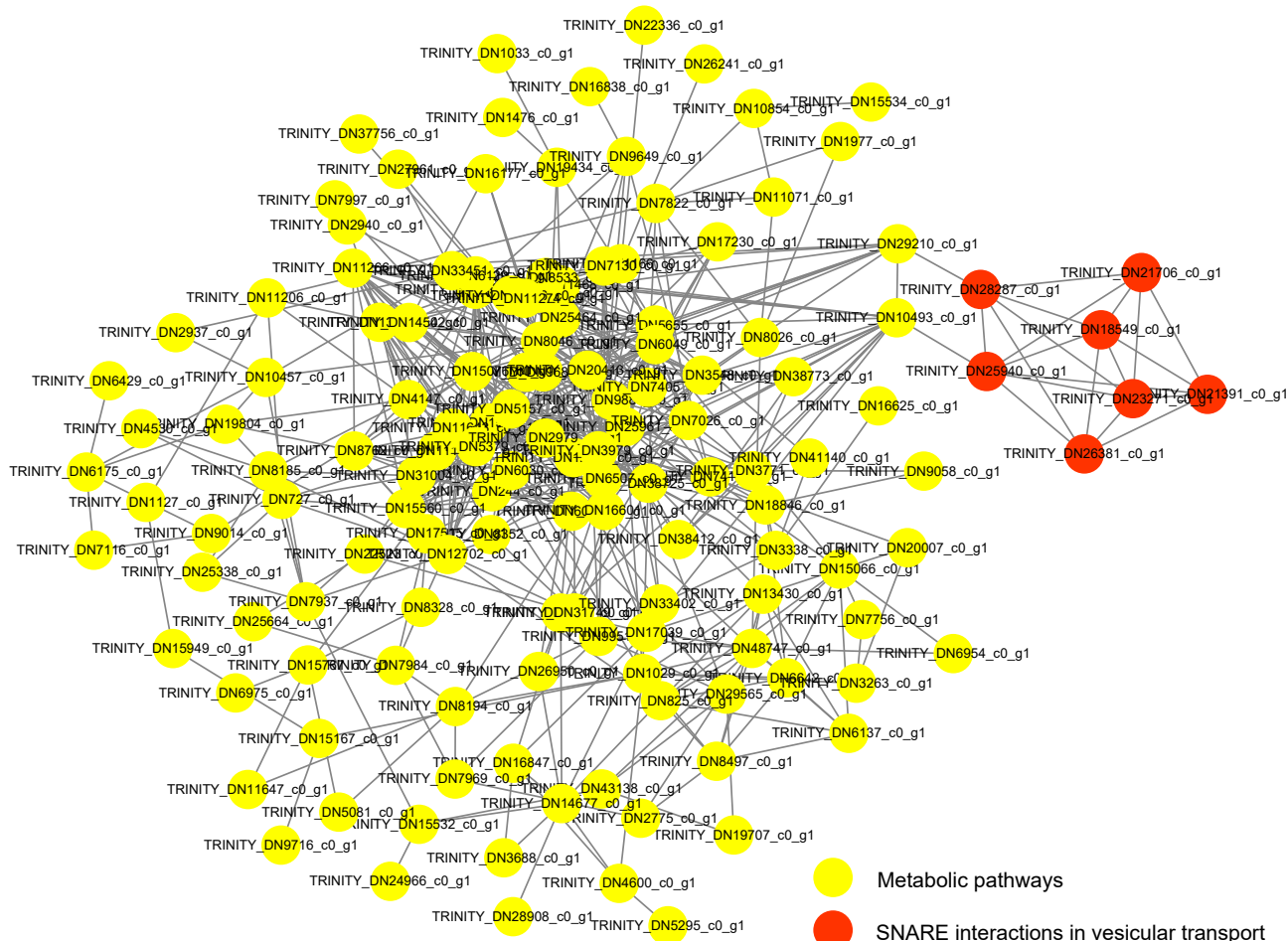

Supplement: Supplementary file 1 [file ijms-25-08875-s001.zip › Supplementary Figures/Fig S5.pdf]

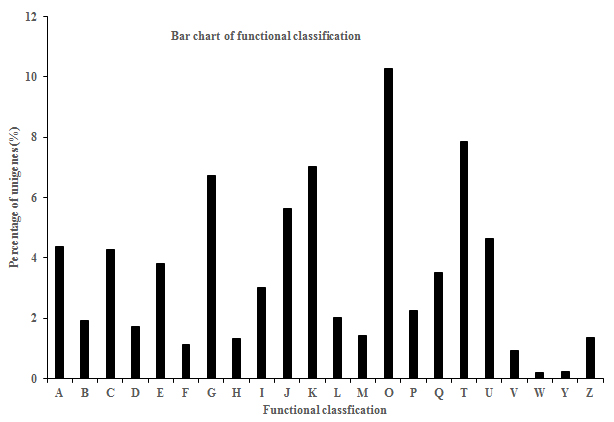

Supplement: Supplementary file 1 [file ijms-25-08875-s001.zip › Supplementary Figures/Figure S1.jpg]

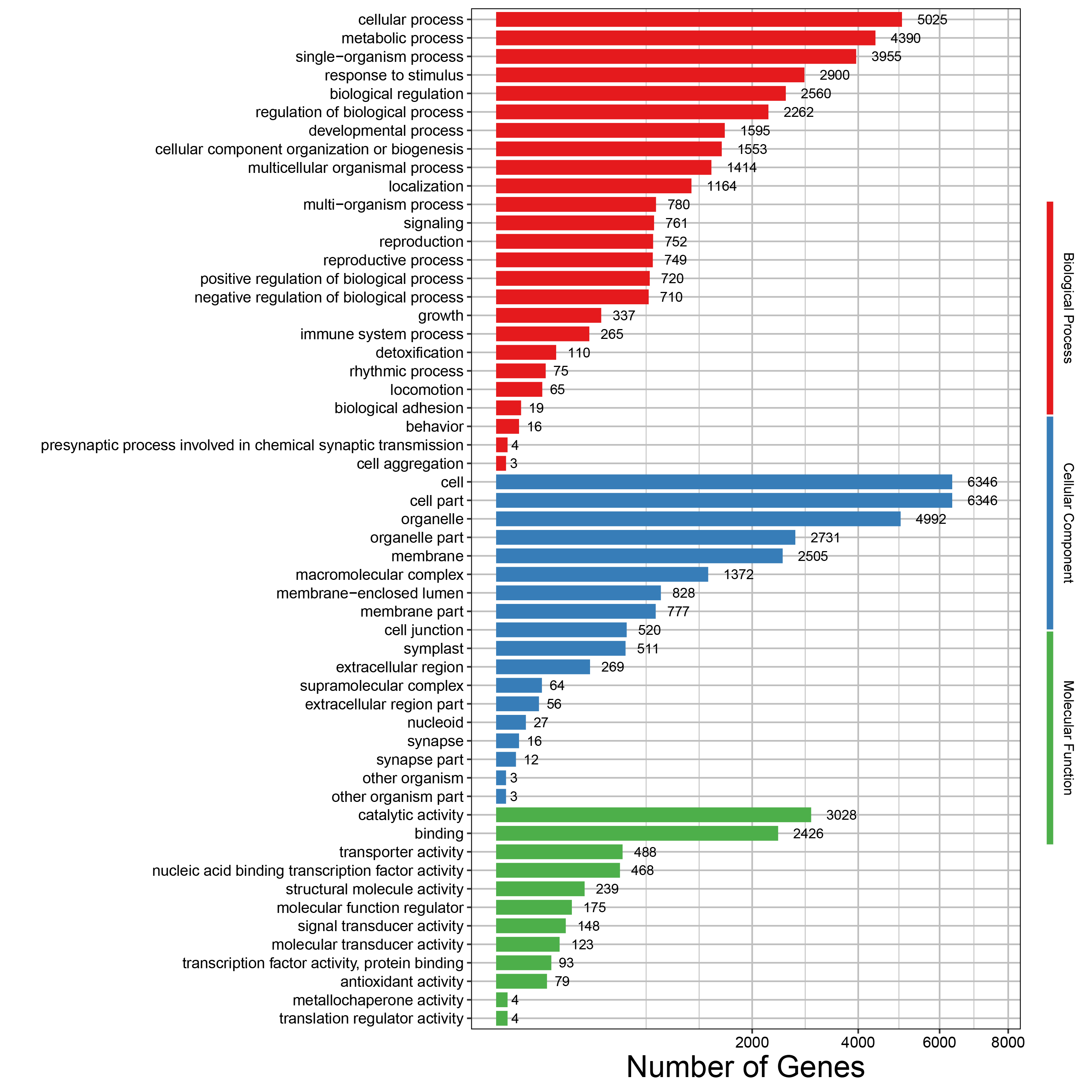

Supplement: Supplementary file 1 [file ijms-25-08875-s001.zip › Supplementary Figures/Figure S2.jpg]

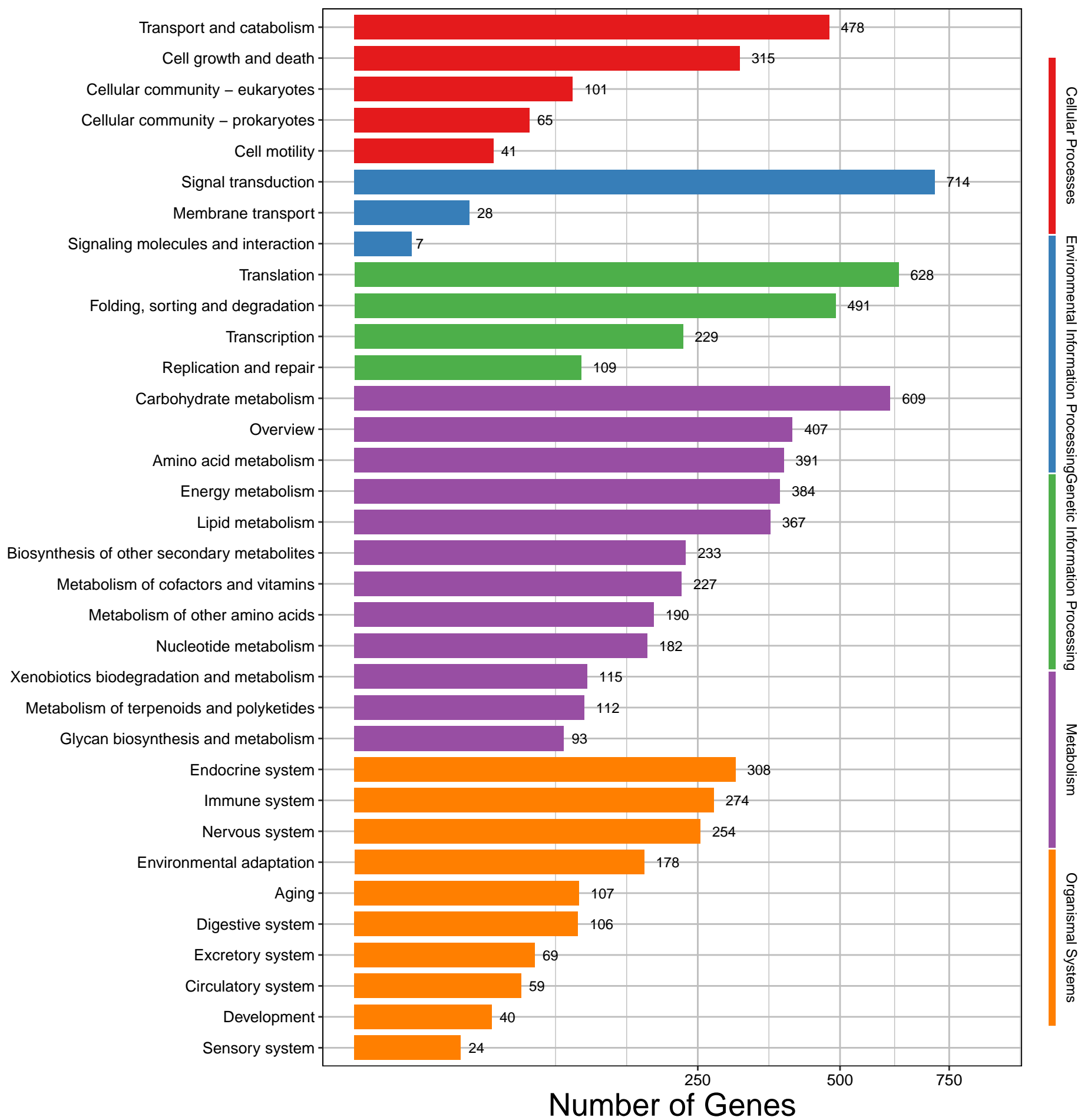

Supplement: Supplementary file 1 [file ijms-25-08875-s001.zip › Supplementary Figures/Figure S3.pdf]

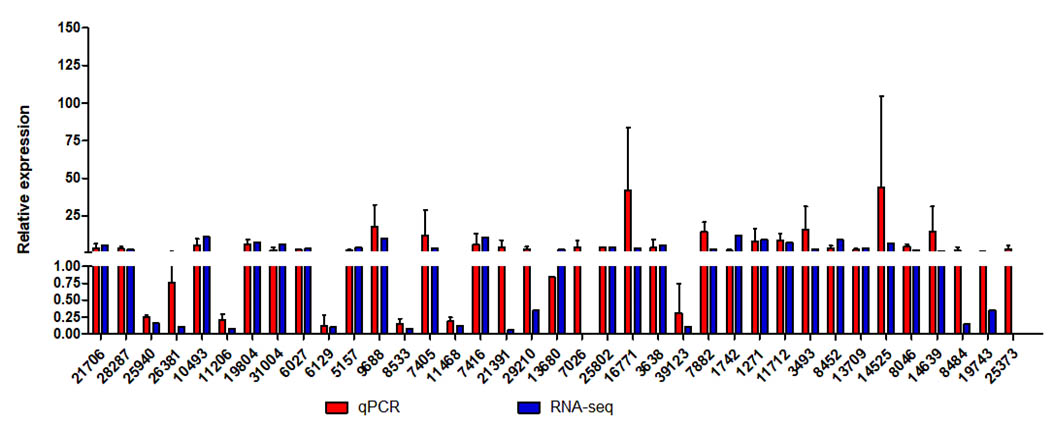

Supplement: Supplementary file 1 [file ijms-25-08875-s001.zip › Supplementary Figures/Figure S6.jpg]
